# Early objective response may not be a prognostic factor of survival for patients with metastatic urothelial carcinoma: from a retrospective analysis of a cohort of 113 patients

**DOI:** 10.1186/s12952-015-0037-5

**Published:** 2015-11-10

**Authors:** Guilhem Roubaud, Véronique Brouste, Phillipe Beuzeboc, Aude Fléchon, Diego Tosi, Sandrine Lavau-Denes, Christine Chevreau, Stéphane Culine, Stéphane Oudard, Amandine Quivy, Philippe Pourquier, Nadine Houédé

**Affiliations:** Department of Medical Oncology, Institut Bergonié, 229, Cours de l’Argonne, 33076 Bordeaux, France; Institut Bergonié, Clinical and Epidemiological Research Unit, 33000 Bordeaux, France; Department of Medical Oncology, Institut Curie, 25 rue d’Ulm, 75005 Paris, France; Department of Medical Oncology, Institut Léon Berard, 69000 Lyon, France; Department of Medical Oncology, Institut Val d’Aurelle, 34000 Montpellier, France; Department of Medical Oncology, CHU Dupuytren, 87000 Limoges, France; Department of Medical Oncology, Institut Claudius Regaud, 31000 Toulouse, France; Centre Hospitalier Universitaire Saint Louis, 75010 Paris, France; European Hospital Georges Pompidou, 75015 Paris, France; Institut de Recherche en Cancérologie de Montpellier & Université de Montpellier 1, INSERM U896, F-34000 Montpellier, France; Department of Medical Oncology, CHU Caremeau, 30029 Nîmes, France

**Keywords:** Cisplatin-based regimen, Metastatic urothelial carcinoma, Objective response, Prognostic factors, Survival

## Abstract

**Background:**

This study aims to better define prognostic factors for patients with metastatic urothelial carcinoma (mUC), and to identify patients who will benefit from first-line cisplatin-based chemotherapy. We test the hypothesis that early objective response (EOR), defined as the occurrence of an objective response following 2 or 3 courses of chemotherapy, could be a prognostic factor for overall survival (OS) and thus be used to guide treatment decisions. Data from 113 patients with evaluable mUC receiving first-line cisplatin-based treatment between January 2004 and December 2006 was collected retrospectively from prospectively-maintained databases across seven French cancer centers. Clinical factors potentially associated with survival and EOR were analyzed in univariate and multivariate analysis.

**Results:**

One hundred three patient records were complete and available for inclusion in the multivariate model. Four factors were independently associated with OS: Performance status 1 and 2 (HR 2.3 [95 % CI 1.3–3.9], *p* = 0.002; HR 3.4 [95 % CI 1.6–7.2], *p* = 0.001 respectively); presence of visceral metastases (HR 2.2 [95 % CI 1.3–3.9], *p* = 0.004); abnormal hemoglobin levels (HR 1.7 [95 % CI 1.01–2.8], *p* = 0.045); disease progression (HR 10.1 [95 % CI 4.2–24.1], *p* < 0.001).

**Conclusions:**

This study confirms the prognostic factors previously reported in first-line chemotherapy for mUC. However, we failed to demonstrate that EOR was an independent predictive factor of OS. Nevertheless, an early response evaluation is recommended since early progression is an important parameter that can be used to decide whether treatment should be interrupted and changed for alternative strategies integrating the concept of personalized medicine or new immune therapies.

## Background

Bladder cancer is the second leading cause of death in urologic cancer in men and women [1]. Cisplatin-based chemotherapy represents the standard treatment for metastatic urothelial carcinoma (mUC) when patients are eligible. Median overall survival (OS) after this regimen has remained relatively stable since the last two decades at around 14–15 months [2, 3]. A better safety profile is obtained when a combination of gemcitabine and cisplatin (G-C) [4], is used as compared to the methotrexate-vinblastine-adriamycine-cisplatin regimen (M-VAC). Vinflunine is the only second-line treatment approved at present, with a small benefit on survival when adjusted on prognostic factors [5] and a restricted availability due to financial reasons. Currently, no other standard treatments are approved after failure of a cisplatin-based regimen, including targeted or immune therapies [6], however recent studies have shown promising results using well tolerated drugs with prolonged clinical benefit [7, 8]. Because of the relatively high toxicity of this first-line regimen, it appears crucial to better identify patients who will benefit the most from this treatment. In practice, if a patient does not respond at the first evaluation after 2 or 3 courses, research is needed to answer the question of whether this regimen should be continued or not.

Prognostic factors of survival and predictive factors of response are necessary to help physicians make clinical decisions. Previous studies have reported prognostic factors of survival based on standard first-line chemotherapies with ECOG Performance Status (PS) and visceral metastasis independently associated with OS in most studies [2, 3, 9, 10]. PS and visceral metastasis are defined as the main independent predictive factors of response to cisplatin-based chemotherapy in mUC [2, 10, 11]. Furthermore, Sengelov *et al.* [11] proposed to identify the impact of objective response (WHO criteria) on survival, analyzing data from 119 evaluable patients (4 consecutive phase II studies performed between 87 and 97) with locally-advanced or mUC at diagnosis, as well as with metastatic or regional relapse after a local treatment. Median survival was 12.4 months in responder patients and 6.3 months in non-responder patients. Response to chemotherapy was included in the multivariate analysis and was found as the strongest prognostic factor for survival. To our knowledge this is the only study that has identified objective response as an independent prognostic factor. We thus aimed to focus on early objective response (EOR), defined as the occurrence of an objective response following 2 or 3 courses of cisplatin-based chemotherapy, hypothesizing that EOR could independently impact on OS. This data could be useful to inform treatment decisions, for example switching protocols for patients with stable disease (SD) and avoiding serious side effects of these treatments that could potentially be inefficient. This treatment strategy could accelerate inclusions in subsequent clinical trials guided by personalized medicine, or new immunotherapies.

## Results

### Population

Medical records for 113 patients with a metastatic urothelial cancer (mUC) were retrieved from prospectively-maintained institutional databases (Table 1). Thirty patients received their first course of chemotherapy in 2004, 40 in 2005 and 43 in 2006. The female/male sex ratio was 1/4.2 and median age at the first course was 63.7 years (range 33.7–79.6). A majority (85 %) of patients had PS of 0–1. Forty-one patients (36.3 %) relapsed following treatment of a localized tumor (with a median time to relapse of 9.47 months) and 72 (63.7 %) had metastatic disease at the time of diagnosis. Eighty-six (76.1 %) patients received a combination of G-C, 12 (10.6 %) an M-VAC regimen, and 15 (13.3 %) had a switch from cisplatin to carboplatin during therapy (in the subset G-C regimen only), after a minimum of 3 cycles of cisplatinum-based regimen for all patients.

**Table 1:** Population characteristics for patients with metastatic urothelial carcinoma treated by first-line cisplatin-based chemotherapy (*n* = 113)

Characteristics	n (%)
Male/female	91 (80.5) / 22 (19.5)
Age in years, (median [range])	63.24 [33.7–79.7]
Performance Status	
0	46 (40.7)
1	50 (44.2)
2–3	17 (15.0)
Metastatic relapse	
Yes	41 (36.3)
No	72 (63.7)
Number of metastatic sites	
1	39 (34.5)
2	51 (45.1)
3	17 (15)
>3	6 (5.3)
Metastatic sites (non-exclusive)	
Lymph node under the diaphragm	84 (74.3)
Lymph node above the diaphragm	20 (17.7)
Bone	36 (31.9)
Lung	44 (38.9)
Liver	23 (20.4)
Other	8 (7.1)
Visceral metastases (lung – liver – bone)	77 (68.1)
Haemoglobin level	
>11g/dl	61 (54)
<11g/dl	42 (37.2)
Unknown	10 (8.8)
Creatinine clearance	
Normal	76 (67.3)
Abnormal	20 (17.7)
Unknown	17 (15)
AP	
Normal	48 (42.5)
Abnormal	32 (28.3)
Unknown	33 (29.2)
LDH	
Normal	36 (31.9)
Abnormal	24 (21.2)
Unknown	53 (46.9)
Chemotherapy	
G-C	86 (76.1)
M-VAC	12 (10.6)
G-C switched to G-Carboplatin	15 (13.3)
Response following 2 or 3 cycles	
CR	3 (2.7)
PR	61 (54)
SD	38 (33.6)
PD	11 (9.7)
EOR	64 (56.7)
Subsequent treatments^a^	51 (45.1)
Chemotherapy	46 (40.7)
Chemoradiotherapy	5 (4.4)
Radiotherapy	0

LDH: lactate deshydrogenase ; AP: alkaline phosphatase ; G-C: gemcitabine – ciplatin ; M-VAC: methotrexate – vinblastine – adriamycine – cisplatin ; G: gemcitabine ; CR: complete response. PR: partial response SD: stable disease. PD: progressive disease. EOR: early objective response (RECIST criteria v.1)

^a^unknown: 20 (25 %)

### Early objective response (EOR)

All patients were available for response evaluation following 2 or 3 cycles. Three patients achieved CR (following 3 cycles), 61 patients achieved PR (23 after 2 cycles and 38 after 3), giving 64 patients with an EOR and an EOR rate of 56.6 %. Thirty-eight (33.6 %) stable disease (SD) and 11 (9.7 %) progressive disease (PD) were reported after 2 or 3 cycles of first line chemotherapy.

### Toxicities, further treatments and survival

Toxicities are reported in Table 2. In terms of Grade 3/4 severe toxicities, neutropenia was the most frequent, followed by thrombopenia and infection. Fifty-one (45.1 %) patients received treatments following and/or completing this first-line chemotherapy with 5 receiving concomitant radio-chemotherapy (no radiotherapy alone) and 46 receiving subsequent chemotherapy.

**Table 2:** Toxicity (NCI CTCAE v.3)

	Grades n (%)			
	1	2	3	4
Fatigue	43	26	12	0
Nausea/vomiting	27	32	8	0
Infection	2	14	12	2
Anaemia	34	33	11	0
Thrombopenia	18	13	13	3
Leucopoenia	9	12	18	11
Renal	21	11	2	0

**Fig. 1:**
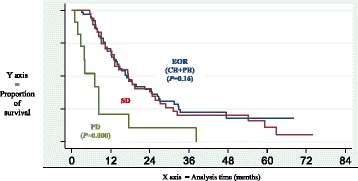
Survival curves according to early objective response (EOR), stable disease (SD) and progressive disease (PD). EOR (blue): early objective response (complete response + partial response; RECIST criteria v.1) SD (red): stable disease, PD (green) progressive disease

**Fig. 2:**
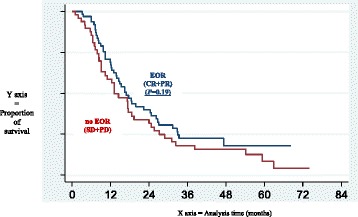
Survival curves according to occurrence or absence of early objective response (EOR). EOR (blue): early objective response (complete response + partial response; RECIST criteria v.1); absence of EOR (red): stable disease + progressive disease

The median follow up was 47.1 months [95 % CI: 41.4–54.3]. At the time of analysis 19 (16.8 %) patients were alive, 85 (75.2 %) had died from their disease, 1 (0.9 %) from treatment-related toxicity and 1 from another cause. Seven (6.2 %) patients were lost to follow-up. The median overall survival was 16.43 months [95 % CI: 13.0–18.6]. Survival according to whether the patients achieved an EOR or not are presented in Figs. 1 and 2. We did not find any significant differences in overall survival according to response (responder *n* = 64 and non-responder *n* = 49): median survival of 16.0 months [95 % CI: 12.9–24.6] and 13.2 months [95 % CI: 9.1–18.6] respectively (*P* = 0.16).

### Prognostic analysis: univariate and multivariate analysis (Table 3)

**Table 3:** Univariate and multivariate analysis of prognostic factors of overall survival for patients with metastatic urothelial carcinoma treated by first-line cisplatin-based chemotherapy (*n* = 103)

	Log rank test	Univariate analysis	Multivariate analysis
		HR [95 % CI] *p* de wald	HR [95 % CI] *p* de wald
Sex	0.88		
Male		0.96 [0.57–1.6] 0.88	
Female		1	
Age	0.34		
<64 years		1	
> = 64 years		0.82 [0.54–1.24] 0.34	
PS	<0.001		
PS 0		1	1
PS 1		2.11 [1.31–3.40] 0.002	2.3 [1.3–3.9] 0.002
PS 2 or 3		4.98 [2.61–9.49] <0.001	3.4 [1.6–7.2] 0.001
Hb level (cut-off: 11g/dl)	0.03		
Normal		1	1
Abnormal		1.65 [1.05–2.6] 0.029	1.7 [1.01–2.8] 0.045
Metastatic relapse	0.90		
No		1	
Yes		0.97 [0.62–1.51]0.90	
N metastatic site	<0.001		
1		1	excluded *p* = 0.76
2		1.63 [0.99–2.70] 0.054	
≥3		2.87 [1.60–5.2] <0.001	
Visceral metastases	<0.001		
No		1	1
Yes		2.29 [1.4–3.7] 0.001	2.2 [1.3–3.9] 0.004
Chemotherapy regimen	0.18		
G-C		1	
M-VAC		0.60 [0.28–1.27] 0.18	
G-C and G-carboplatin		1.40 [0.77–2.55] 0.27	
Response to treatment	0.002		
SD		1	1
EOR (CR + PR)		0.93 [0.6–1.5] 0.75	1.45 [0.9–2.4] 0.16
PD		3.2 [1.5–6.5] 0.002	10.1 [4.2–24.1] 0.000

Five parameters were found to be related to OS following univariate analysis: higher PS (*P* = 0.001*),* more metastatic sites (*P* < 0.001), presence of visceral metastases (*P* < 0.001), abnormal hemoglobin level (*P* = 0.029), and the occurrence of an EOR (*P* = 0.002).

Ten patients (8.8 %) were excluded from the multivariate analysis due to incomplete hemoglobin level data. Four factors were independently associated with poorer OS: PS 1 (HR 2.3 [95 % CI 1.3–3.9], *p = 0.002)* and PS 2 or 3 (HR 3.4 [95 % CI 1.6–7.2], *P* = 0.001*)*, presence of visceral metastases (HR 2.2 [95 % CI 1.3–3.9], *P* = 0.004*)*, abnormal level of hemoglobin (HR 1.7 [95 % CI 1.01–2.8], *P* = 0.045), and progressive disease, (HR 10.1 [95 % CI 4.2–24.1], *P* < 0.001). The hazard ratio for EOR (CR + PR) was 1.45 [95 % CI 0.9–2.4], *P = 0.16*.

## Discussion

This multicenter retrospective study identified four independent prognostic factors of poorer overall survival: high PS, presence of visceral metastases, abnormal levels of hemoglobin and early PD. All patients had metastatic disease (not locally advanced) and were initially treated with a cisplatin-based regimen corresponding to an homogeneous population. Moreover, it represents a good reflection of practice in France, with a majority of patients being treated with a G-C regimen instead of M-VAC as first-line chemotherapy for mUC. Patient characteristics and median OS are similar to other studies [4, 9–15]. Toxicity was comparable to that reported in the phase III study by von der Maase *et al.* [4].

PS and visceral metastases are well known as independent prognostic factors of survival in mUC and have been previously reported in at least 10 different studies [2, 3, 9–13, 16–18]. Early PD was an independent prognostic factor of survival and EOR was not. Our data did not confirm that previously reported by Sengelov *et al.* [11] regarding the impact of objective response on survival. This could be explained by several differences such as period of treatment and care management (between 1987 and 1997 vs. between 2004 and 2006), assessment of objective response (WHO vs. RECIST criteria), or time of assessment (not specifically following 2 or 3 cycles, in the first step of treatment). Unlike Sengelov *et al.* we preferred to maintain three groups in our analysis (EOR, SD and PD), considering the absence of significant differences in survival between the responder population and those with stable disease (*P = 0.16* in multivariate analysis and in Fig. 1). Survival curves have also been presented by Sengelov *et al*. [11] and seem to show a comparable survival between SD and PR (median OS not shown by the authors). To go further in the comparison, in Fig. 2, using the same presentation as Sengelov *et al.* with OS according to responder *versus* non responder patients, we did not find any significant differences between the two groups (Fig. 2). Nevertheless, this methodology of separation of categories previously reported could be discussed especially for SD and PR populations, which seem to be comparable across the two studies.

Our report contains some biases, which are inherent to any retrospective study over a long period. First, the chemotherapy protocol is not homogeneous, as different regimens were used including a switch from a cisplatin- to carboplatin-based regimen for 13.3 % of patients during therapy. This regimen is used for the “unfit population” for a cisplatin regimen and median overall survival reported is approximately 10 months, which is less than other reports using cisplatin-based regimen [14]. However in these subsets, no differences were reported between GC, M-VAC and GC switched to G-Carboplatin in univariate analysis on survival (*P* = 0.18). Second, survival data could be influenced by subsequent treatments, such as concomitant chemo-radiotherapy or subsequent chemotherapies, which were not tested in our analysis due to their dependence on the results to the chemotherapy. Third, we excluded 10 (8.8 %) observations, leaving 103 patients for multivariate analysis: this relatively low number could have contributed to a lower statistic power as compared with previous reports *e.g.* 119 patients in study reported by Sengelov *et al*. [11].

We did not confirm our primary hypothesis in this study, even if we found that early progression has an impact on survival for this population. EOR could remain a relevant predictive factor of survival since the other well-known parameters were found to be independently associated with OS, and this difference could be explained by a lack of statistical power. This data does not indicate that changes in treatment strategies are required, but it could be useful to predict early progression following standard first-line chemotherapy, and avoid potential side effects from this treatment.

The obvious question now is how to treat patients who will not benefit from cisplatin-based regimens? Previous studies have proposed some sequential chemotherapy in bladder cancer with limited effects reserved to highly selected populations (good performance status and limited metastatic spread) as reported by Siefker Radtke *et al.* [15]. New drugs and progress in tumoral biology understanding could provide alternative treatments [19, 20]. Recent phase II trials using everolimus or temsirolimus [21, 22] and nanoparticle albumin-bound paclitaxel [23] achieved clinical benefit and durable response. Similarly, immune therapies targeting PD-1 and PD L-1 have shown noteworthy results in terms of objective response rate and response duration in phase I recently reported [7, 8]. Thus, it is important to detect the absence of efficacy of a toxic first-line regimen early, to enable a change for a new treatment either in the era of immunotherapy or potentially integrated in a personalized medicine approach according to well-known biomarkers in urothelial carcinoma [24]. An interim analysis of the prospective trial MOSCATO 01 has shown feasibility in daily practice of such an approach [25].

## Conclusions

In conclusion, this multicenter French retrospective study performed on a population of patients treated with cisplatin-based regimen for a mUC, does not support the hypothesis that EOR could be associated with a better survival. Nevertheless, an early response evaluation after 2 or 3 courses of chemotherapy is recommended because early progression is an independent prognostic factor of poorer survival, indicating that the treatment should be stopped or changed earlier, favoring new strategies based on personalized medicine or immune therapies.

## Methods

This retrospective multicenter study involving seven French medical centers was approved by the French data protection authority (Commission Nationale Informatique et Liberté, CNIL) and was supported by the French cooperative GU tumors group (GETUG). According to French data protection authority rules, a patient informed consent was not required, however, a document of information was provided to patients who were alive at the time of the analysis. Data was collected from the medical files of patients including patient characteristics, survival data and clinical parameters previously described as independent prognostic and/or predictive factors (sex; age (<64/> = 64y); PS (0,1,2 or 3); Hemoglobin level (normal/abnormal ≤11g/dl); metastatic relapse (yes/no); number of metastatic sites (1,2,> = 3); visceral metastases (defined as presence of metastases in the liver and/or lung and/or bone); chemotherapy regimen (G-C, M-VAC, G-C and G-carboplatin), all patients had cisplatin based regimen prior assessment of response following 2 or 3 cycles. An EOR was defined as the occurrence of partial response (PR) or complete response (CR) obtained following 2 or 3 courses of chemotherapy, according to RECIST criteria 2000 [16]. Stable disease was used as a reference category comparing SD and EOR. Only patients with metastatic (all T, all N and M1 according to the TNM classification 2002) urothelial carcinoma were included. All patients had not received chemotherapy prior to being treated with either G-C or M-VAC and treatment started between January 1^st^ 2004 and December 31^st^ 2006. Toxicity data was collected in the clinical chart and classified retrospectively according to the NCI CTCAE v.3 scale. Statistical analyses were performed with SPSS v.17 software (Cary, NJ). Survival probability was calculated by the Kaplan-Meier method, and defined as the time from the first day of the initial course of chemotherapy to the date of last follow-up or death. Median follow-up was calculated by the reverse Kaplan-Meier method and is reported with 95 % confidence interval (CI). Univariate analyses of prognostic factors and EOR was performed using log rank tests and all variables significant at *P* < 0.05 were included in the multivariate analysis. After a test of proportional hazard assumptions, the multivariate model used a stepwise descending method and *P* < 0.05 was considered significant.
